# Biocompatibility of new bioactive resin composite versus calcium silicate cements: an animal study

**DOI:** 10.1186/s12903-019-0887-1

**Published:** 2019-08-22

**Authors:** Ashraf Abou ElReash, Hamdi Hamama, Walied Abdo, Qiqi Wu, Ahmed Zaen El-Din, Xie Xiaoli

**Affiliations:** 1 Department of Endodontic, Xiangya School of stomatology, Central South University, Xiangya Road No 72. Kaifu, Changsha, 410078 Hunan province China; 2 Department of Operative Dentistry, Faculty of Dentistry, Mansoura University, Mansoura, Egypt; 3 Department of Pathology, Faculty of Veterinary Medicine, Kafrelsheikh University, Kafrelsheikh, Egypt; 4 Department of Conservative Dentistry. Faculty of Oral and Dental Medicine, Delta University for Science and Technology, International Coastal Road, Gamasa City, Mansoura, Dakhliya Egypt

**Keywords:** Bioactive composite, Calcium silicate, Caspase 3, MTA-HP, α-SMA

## Abstract

**Background:**

The purpose of this study was to compare the biocompatibility of three bioactive materials, namely ACTIVA bioactive restorative resin composite, iRoot BP plus and Mineral Trioxide Aggregate (MTA) Angelus-HP.

**Methods:**

Seventy-five Wistar male rats were subjected to subcutaneous implantation of four polyethylene tubes; one empty tube was used as control (Group 1), and the other tubes were filled with ACTIVA (Group 2), iRoot BP (Group 3) and MTA-HP (Group 4). Then, the rats were subdivided into 3 groups according to the sacrification time into one, two and 4 weeks (*n* = 25 rats). Tissue specimens were submitted to histopathological and immunohistochemical analysis of α-SMA and caspase 3.

**Results:**

The one-way Anova test revealed that ACTIVA group exhibited minimal inflammation in comparison to calcium silicate cements (iRoot BP and MTA-HP groups). iRoot BP group significantly revealed a more severe degree of chronic inflammation in comparison to other groups (*P* < 0.05). ACTIVA group showed marked regression of inflammation and fibrosis comparable to the control, while iRoot BP group revealed remarkable fibrosis and calcification, with less degrees in MTA-HP group (*P* < 0.05). Immunostaining of both α-SMA and caspase 3 revealed lower indexes in ACTIVA group consistent with the control (*P* < 0.05).

**Conclusions:**

ACTIVA showed a higher degree of biocompatibility to subcutaneous tissues in comparison to both iRoot BP and MTA-HP cements in regard to decrease the intensity of inflammation, with subsequent fibrous connective tissue remodeling and better healing patterns.

**Clinical significance:**

Preliminary data suggests that the application of ACTIVA in retrograde fillings.

## Background

Endodontic procedural errors may include traumatic pulp exposure and root perforations. Subsequent communication with surrounding periapical tissues may occur, resulting in a challenging situation for operators. A number of different bioactive repair materials can be used to seal these perforations [1]. Biocompatibility and tissue tolerance without election of inflammatory responses are essential prerequisites for a successful endodontic repair material as it comes in direct contact with living human bones, periapical tissues and body fluids [2]. From this point on, finding a suitable material to substitute the lost tooth structure and obtain successful outcomes in such clinical situations, becomes crucial [1].

Mineral Trioxide Aggregate (MTA) provides a great solution due to it’s high biocompatibility, ability to set in presence of water, good sealing ability, bioactivity and other desirable properties for clinical applications [3–5]. Despite of its advantages, the disadvantages of MTA include discoloration, long setting time, low flowability and handling difficulties. This led to the need of improvement in physical properties and the development of an enhanced formula to overcome these drawbacks [6].

In 2002, a second generation known as “white MTA” was introduced to the dental markets with smaller and more even particle distributions in order to overcome the drawbacks of grey MTA [7]. MTA Angelus HP (Londrina, PR, Brazil) was introduced with better handling properties and higher plasticity as the manufacturer added an organic plasticizer to the liquid. The manufacturer also replaced bismuth oxide by calcium tungstate as radiopacifier to avoid teeth discoloration. Moreover, the elimination of arsenic and a reduced setting time (15 min) was also accomplished, compared to other MTA based materials [8–10].

iRoot BP Plus Root Repair Material (BP-RRM; Innovative BioCeramix Inc., Vancouver, BC, Canada), is a new fully synthetic laboratory material using bioceramic nanotechnology, which is introduced as a ready-to-use premixed bioceramic paste mainly composed of calcium silicate- based compounds. It hardens in the presence of water and needs a minimum of 2 h to completely set. As claimed by the manufacturer, its main advantages are the fact that it is presented in a readymade paste, requiring no mixing, low solubility, high radiopacity, low setting shrinkage and excellent physical properties [11, 12].

The widespread use of composite as root end filling materials is probably hindered by its technique sensitive nature and the requirement for a good moisture control to guarantee a successful adhesion process. Moreover, it may cause local inflammation when it comes in contact with peri-radicular tissues [13]. ACTIVA bioactive composite (Pulpdent, Watertown, MA, USA), as the manufacturer claims, is the first bioactive resin composite material stimulating mineralization at tooth restoration interface with resilient ionic resin matrix [14]. While the approval document obtained from the US Food and Drug Administration (510 k123265) described ACTIVA as self-adhesive dual cured resin modified glass ionomer (RMGI), ACTIVA is claimed to combine the water friendly properties, more release and recharge of calcium, phosphate and fluoride ions than glass ionomers with the durability and improved physical properties of resin- based materials [15].

Although the wide use of these materials as bioactive resin composite for cavity restoration, a potential use may be as a retrofilling materials. To date, no information regarding ACTIVA placed in contact with subcutaneous tissues is reported in literature. Thus, the aim of this study was to compare the biocompatibility of MTA angelus HP, iRoot BP plus and ACTIVA bioactive restorative in terms of inflammatory response, apoptotic activity and healing ability by subcutaneous tissue implants in rats. The current study was designed to test the null hypothesis where there was no difference in the biocompatibility between the three materials.

## Methods

The tested materials are listed in Table 1.

**Table 1:** Materials used for the study

Materials	Constituents	Manufacturer
Mineral trioxide aggregate High Plasticity (MTA-HP)	Powder: BI_2_O_3_, CaO, MgO, K_2_O, Na_2_O, Fe_2_O3, SO_3_, SiO_2_, Al_2_O_3_ Liquid: distilled water with organic plasticizer	Angelus Co. Londrina, PR, Brazil
iRoot BP Plus	Paste containing: Calcium silicates, zirconium oxide, tantalum pentoxide, calcium phosphate monobasic	Innovative BioCeramix Inc. Canada
ACTIVA Bioactive Restorative	Light curing (resin based) Blend of diurethane and other methacrylates with modified polyacrylic acid (∼53.2%), silica (∼3.0%), sodium fluoride (∼0.9%), and so on	Pulpdent, Watertown, MA, USA

### Animal study and design

This study was conducted after being approved by the ethical committee of Central South University for animal experiments (No: 20180025). Seventy-five male rats (*Rattus norvegicus* albinus, Wistar) weighing approximately 180–200 g provided by Institute of Basic and Clinical Pharmacology Research Centre of Hunan Medical University were used according to the ARRIVE guidelines (Animal Research: Reporting of In Vivo Experiments, 2013) and the procedures conformed to the requirements of the ISO 10993-1 (1992) and ISO 10993-2 (1992) standards [16–18]. Animals were kept in an aerated chamber with 12-h dark and light intervals, divided according to study period. The cages were cleaned on a daily basis, and free access to food and water was allowed. Animals were then given water only 12 h before surgery.

**Fig. 1:**
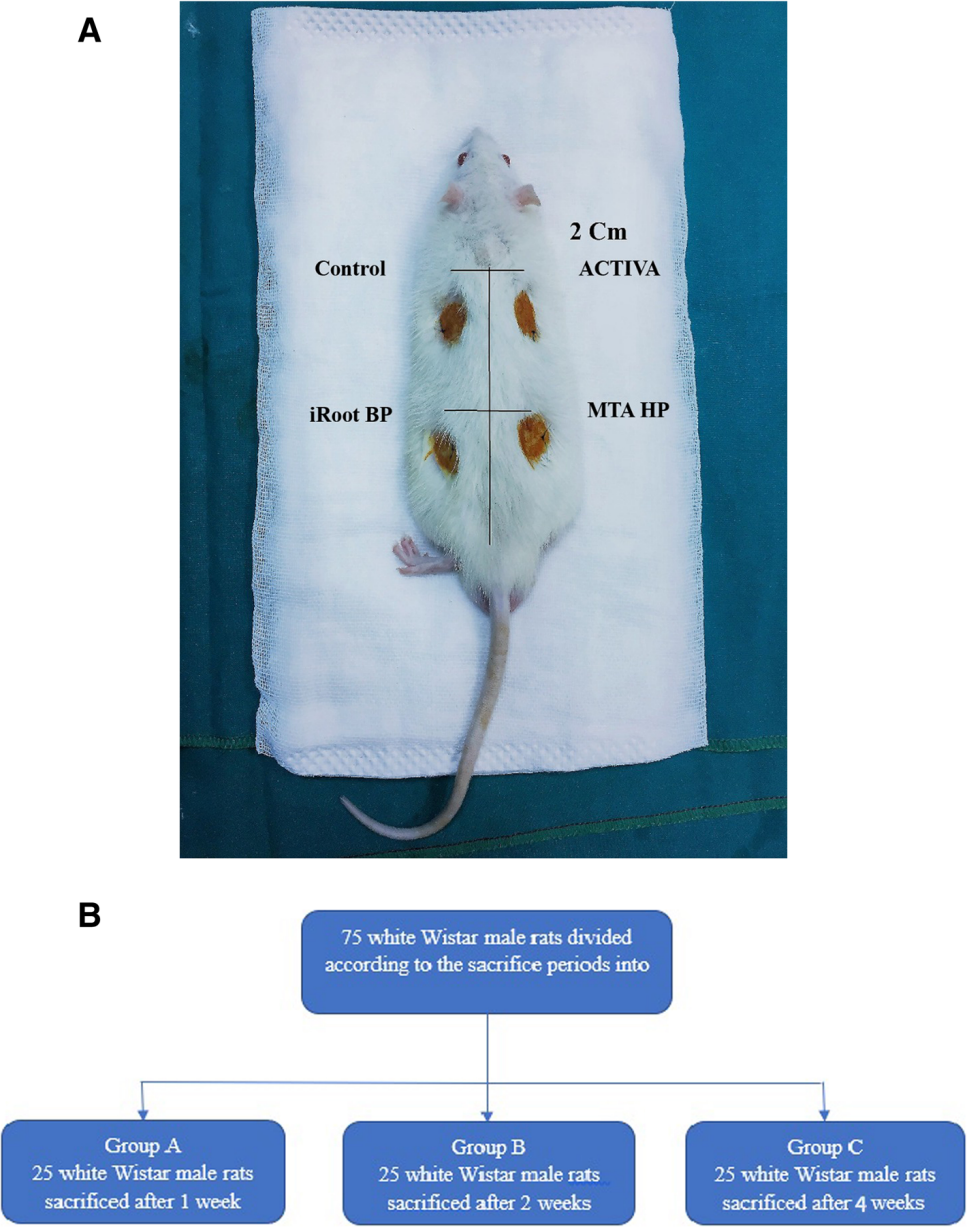
**a** Photograph illustrating the four groups of materials implanted in the dorsal region underneath the skin of each rat. **b** diagram illustrating the classification of groups according to scarification time

Three hundred sterile polyethylene tubes (10 mm in length and 1.3 mm in diameter) were used and filled with the test materials then implanted in the dorsal region underneath the skin of each rat which were divided into 4 groups: (Fig. 1a)

1. **Group 1** (**Control,** *n* = 75 tubes): Empty polyethylene tubes.
2. **Group 2** (**ACTIVA,** *n* = 75 tubes): Polyethylene tubes filled with ACTIVA (Pulpdent, USA).
3. **Group 3** (**iRoot BP,** *n* = 75 tubes): Polyethylene tubes filled with iRoot BP plus (Innovative BioCeramix Inc., Vancouver, BC, Canada).
4. **Group 4** (**MTA-HP,** *n* = 75 tubes): Polyethylene tubes filled with MTA-HP (Angelus HP, Londrina, PR Brazil).

The materials were manipulated according to manufacturers’ instructions. A small condenser was used to insert the test materials into the tubes. The tubes were entirely filled with the tested materials and left for setting. Before surgery, the animals were anesthetized with ketamine hydrochloride and xylazine hydrochloride Intraperitoneal injection (3, 1 respectively; 0.05 mL/100 g of weight; I/P). The dorsal region of each rat was submitted to trichotomy, and antisepsis was done using a gauze moistened with 0.12% chlorhexidine. The dorsal region was then swapped with a gauze soaked with saline solution to remove any antiseptic residue. Four incisions were performed on the dorsal region of each rat (2 anteriorly and 2 posteriorly). The skin lateral to the incisions was pinched, and subcutaneous dissection was done using blunt-ended scissors.

Each animal received 4 tubes: 2 in the anterior dorsal region (Group1 to the left side and group 4 to the right) and 2 tubes in the posterior region (Group 2 to the left side and group 3 to the right). Then, the incisions were closed with Nylon 3–0 sutures. The rats were further subdivided into 3 groups according to the time period of sacrifice into: Group A after 1 week, Group B after 2 weeks and Group C after 4 weeks. (Fig. 1b) By the end of each period, 25 animals were euthanized by administering an anesthetic overdose with ketamine hydrochloride and xylazine hydrochloride Intraperitoneal injection (1: 1 respectively; 0.15 mL/100 g of weight; I/P). Biopsies of skin and subcutaneous tissues (2 × 2 cm) containing the implants were obtained with 1-cm safety margins.

### Histological procedures

The subcutaneous tissues containing the tubes were excised, fixed in 10% neutral formalin for 48 h. After that, the specimens were trimmed parallel to the tube leaving at least 2 mm of tissue on each side, cut into two equal halves, and the tubes were removed. Then, the specimens were inserted in serial ascending concentration of ethyl alcohol for dehydration, followed by clearance in xylene and embedded in paraffin at 58–62 C°. Samples were casted parallel to the long axis of the tube to show the region of interest (tube opening) then serial sections of 4 μm thickness were prepared for further staining with hematoxylin & eosin stain (H&E).

### Immunohistochemistry

Sections from the prepared paraffin blocks were mounted on super frosted coated glass slides (Fisher Thermo Scientific, Nepean, Ontario, Canada) for immunohistochemistry. The sections were cleared, hydrated and subjected to antigen retrieval in EDTA solution (pH = 8) for 15 min at 37 °C. Subsequent incubation of the sections was performed with primary antibodies against α-smooth muscle actin (Thermo Fisher Scientific, Fremont, CA, USA; Cat. No. A1–70007) and caspase 3 (cleaved form of concentrated polyclonal antibody, CP 229A, B, C, 1:200, rabbit, Biocare medical, Pacheco, USA). The slides were rinsed in phosphate buffer saline and incubated with anti-rabbit IgG secondary antibodies (EnVision + System HRP; Dako) for 30 min at room temperature, visualized with di-aminobenzidine commercial kits (Liquid DAB+Substrate Chromogen System; Dako), and finally counterstained with Mayer’s haematoxylin. The negative control sample was prepared with replacing the primary antibody with normal mouse serum.

### Assessment of histopathological and immunohistochemical sections of α-SMA and caspase 3

Histological examination was performed using Leica Microscope (Leica Microsystems GmbH, Wetzlar, Germany**)** adapted with a DFC420 camera. The histopathological grading was evaluated according to the following criteria: necrosis, inflammatory cell infiltrate (polymorph nuclear and mononuclear cells), congestion, macrophage activity (macrophages and multinuclear giant cells which independently scored), vascularization (blood vessels) and fibrosis (fibroblasts). The previous analysis points were scored as (0) absent, (1) discrete, (2) moderate, and (3) severe. The thickness of the fibrous capsule was measured with ImageJ analysis software (NIH, MD, USA) [19–21]. The scoring system was performed by an experienced blinded pathologist, and scores were organized on specific sheets. Quantitative scoring of α-SMA and caspase 3 immunostaining were done according to determination of threshold basis using ImageJ software and expressed as the percent of positive area /mm^2^.

### Statistical analysis

Data were analyzed using the GraphPad Prism 4.0 Software (GraphPad Software, La Jolla, CA). The mean range difference between experimental groups was assessed using Kolmogorov- Smirnov test, values of α-SMA and caspase 3 obtained were statistically compared with One-way ANOVA (One-way analysis of variance) and then multiple comparisons were done using Tukey’s test (*p* < 0.05) to determine the level of significance.

## Results

### Histopathological findings

**Fig. 2:**
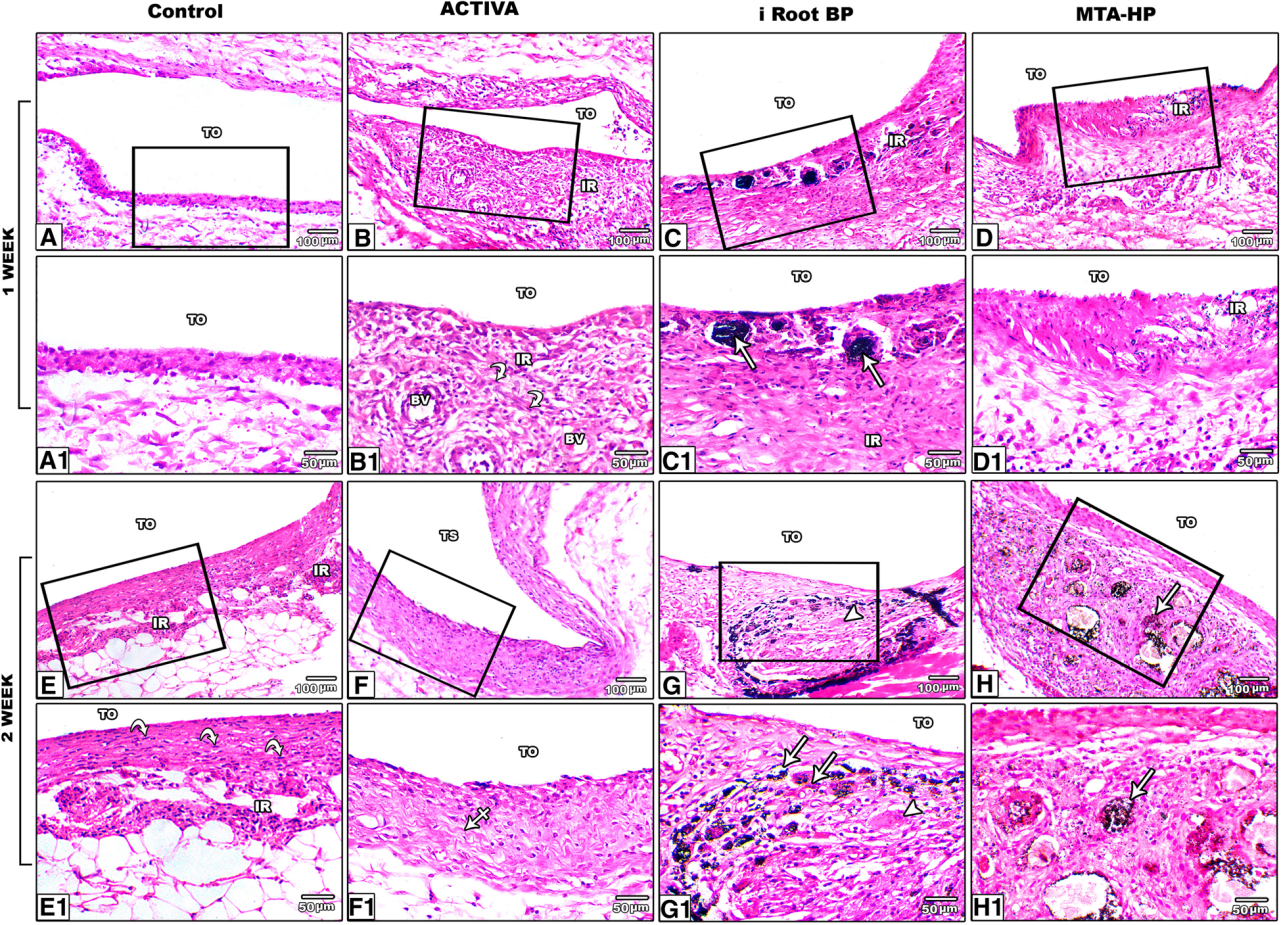
Photomicrograph of H&E staining showing the subcutaneous tissue of control, ACTIVA, iRoot BP and MTA-HP after one- and two-weeks exposure. The 1st week, **a** control tube showing mild inflammatory reaction within and around the capsule, **a1** the inset revealing thin capsule with minimal fibrinoid-like deposition infiltrated with few macrophages; **b** the opening of ACTIVA tube demonstrating moderate degree of inflammation (IR) with marked granulation tissue formation, **b1** the inset showing infiltration of lymphocytes and macrophages and with marked fibroblastic activity (curved-arrow); **c** the iRoot BP tube showing intense inflammatory response, **c1** the inset showing marked fibrosis and calcification within the inflammatory area (arrow); **d** MTA-HP tube showing moderate degree of inflammation, **d1** the inset revealing mild inner necrosis associated with macrophages and lymphocytes infiltration. The 2nd week, **e** Control tube showing an obvious repair of the inflammatory capsule, **e1** inset showing marked remodeling of the fibrous connective tissue (curved-arrow); **f** ACTIVA tube showing marked decrease of necrosis and inflammation **f1** the inset showing ample amount of zigzag-like layers of elastic and collagen fibers (crossed-arrow); **g** iRoot BP tube showing thick capsule with still noticeable intense inflammation **g1** the inset showing fibrosis, calcification (arrow) and multinuclear giant cells infiltration (arrowheads); **h** MTA HP tube showing relatively thick inflammatory capsule, **h1** the inset showing leakage of some tube material associated with inflammatory cells infiltration with mild to moderate fibrosis and calcification (arrow). TO: Tube Opening, IR: Inflammatory Reaction, Bv: Blood vessel

Semi quantitative scoring of the histopathological findings of control, ACTIVA, iRoot BP and MTA-HP was illustrated in Table 2. The statistical analysis was conducted on 60 rats because 15 rats were excluded due to either removal of tube or mortality. On the first week, control group showed thin inflammatory wall, with mild fibrin clot associated with minimal tissue necrosis and few macrophages and neutrophils cells infiltration. Marked tissue organization and angiogenesis around the tube were apparently noticed. Calcification or multinuclear giant cells infiltration were completely absent within the examined animals of this group (Fig. 2a and a1). ACTIVA group revealed slight to moderate inflammatory reaction on the tubular orifice. There was slight fibrinous inflammatory response with a pronounced granulation tissue formation and a mild inflammatory cells infiltration consisted of lymphocytes, macrophages and few neutrophils (Fig. 2b and b1). The subcutaneous tissue reaction to iRoot BP group demonstrated thick inflammatory capsule associated with extensive tissue necrosis, black brownish-leaked deposits of the test material, focal calcification as well as marked fibrosis and angiogenesis especially towards the adjacent connective tissues. The cellular inflammatory response in this group predominately showed macrophages and multinuclear giant cells infiltration (Fig. 2c and c1). MTA- HP group showed thin layer of necrotic tissue admixed with tiny fibrin clotted areas, along with the presence of particulates of brownish pigments especially at the tubular opening. Mild to moderate inflammatory reaction, consisted of mononuclear cells infiltration mainly lymphocytes, macrophages and few neutrophils without any apparent multinuclear giant cells. The fibrosis degree was scanty with immature granulation tissue formation (Fig. 2d and d1).

**Table 2:** Scoring of histopathologic events observed in each group at different time intervals of the study

Histopathological parameters	1 week				2 weeks				4 weeks			
	Control	ACTIVA	iRoot BP	MTA- HP	Control	ACTIVA	iRoot BP	MTA- HP	Control	ACTIVA	iRoot BP	MTA- HP
Coagulative necrosis	1	2	3	2	1	1	3	2	0	0	1	1
Inflammatory response	1	2	3	3	1	1	3	2	0	0	2	1
Vascular reaction	1	1	3	2	1	1	3	2	0	0	1	1
Macrophages	1	1	3	2	1	2	3	2	0	1	2	1
MNGCs	0	0	2	1	0	1	3	2	0	0	1	1
Angiogenesis	1	2	1	1	1	1	3	2	0	1	1	1
Fibrosis	1	1	2	2	1	1	3	2	0	1	2	1
Thickness of fibrous capsule (μm)	142.08±32.79	235.14±27.39	460.99±33.86	352.72±44.45	169.43±39.90	180.90±43.49	410.52±75.69	289.56±62.44	109.89±30.48	132.55±34.79	270.90±56.87	197.93±31.14

Scores: 0, absent; 1, discrete; 2, moderate; 3, severe. *n* = 25

Thickness of fibrous capsule was expressed as mean of values ±SD

On the period of 2 weeks, control group showed an early remodeling of the fibrous connective tissue capsule, mild inflammatory cells infiltration and slight angiogenesis (Fig. 2e and e1). ACTIVA group demonstrated marked decrease in both inflammation and tissue necrosis with regression of inflammatory cells number. The capsule was lined with fibroblast layer, encircled by medial layer of collagen and elastic connective tissues that normally oriented in parallel pattern. The outer layer showed fibrous connective tissue associated with minimal both angiogenesis and inflammatory cells infiltration consisted of macrophages and lymphocytes (Fig. 2f and f1). iRoot BP group showed a noticeable chronic inflammatory reaction at the tube orifice associated with intense fibrosis, calcification and multinuclear giant cells infiltration. Active angiogenesis was markedly increased, not only limited to the tube opening but also extended deeper into the adjacent tissues (Fig. 2g and g1). MTA- HP group revealed similar reaction to the previous group but with a lesser degree regarding the level of calcification, fibrosis and multinuclear giant cells infiltration (Fig. 2h and h1).

**Fig. 3:**
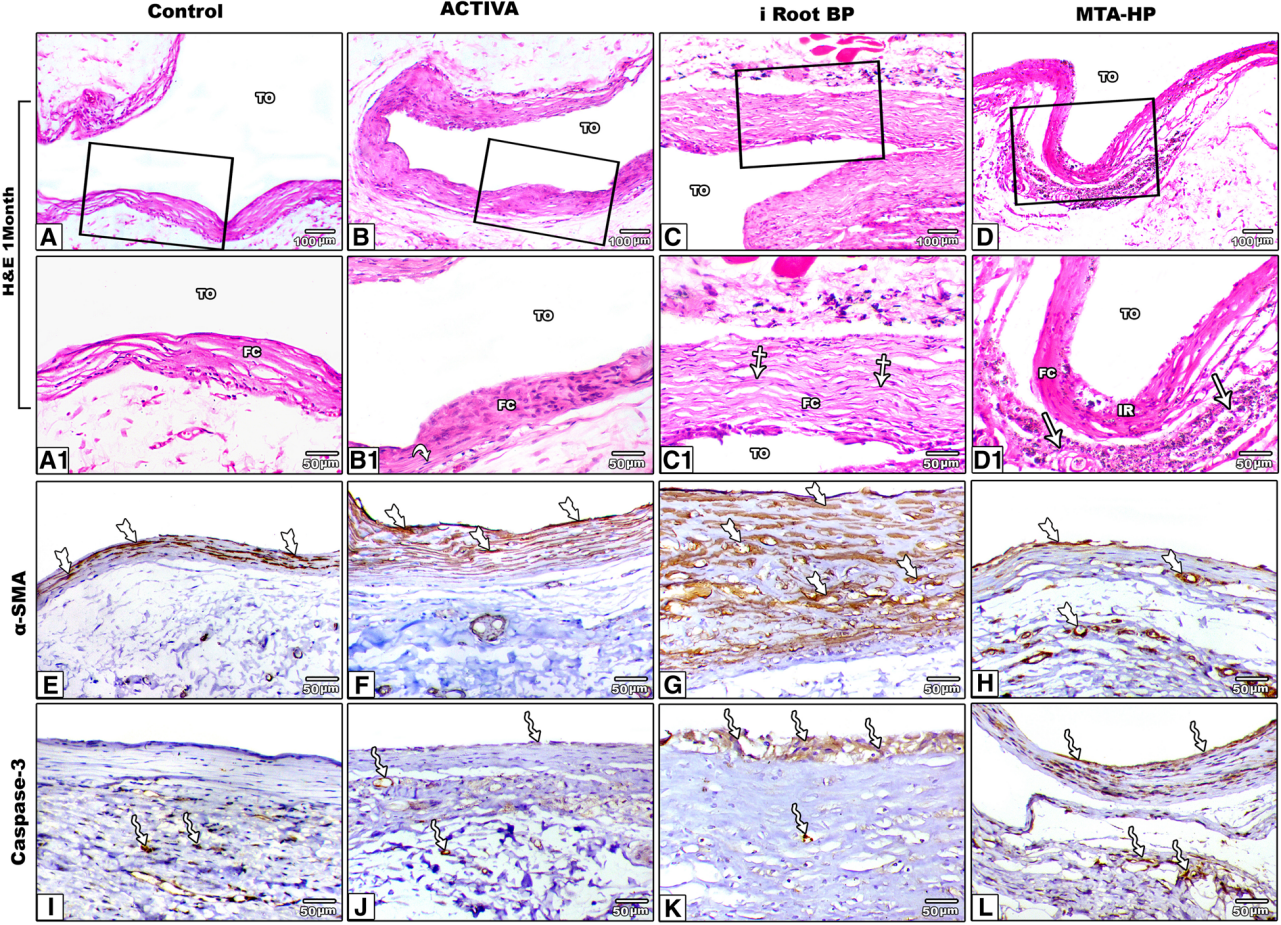
Photomicrograph of H&E staining sections showing the subcutaneous tissue implant of control, ACTIVA iRoot BP and MTA HP after 4 weeks in addition to α-SMA and caspase 3 immunostaining. After 1 month experiment, **a** control tube showing thin fibrous capsule, **a1** the inset revealing normal well organized fibrous connective capsule (FC); **b** ACTIVA tube showing thin fibrous capsule, **b1** the inset showing mild inflammatory cells infiltration, fibroblasts and moderate fibrous connective tissues (curved-arrow); **c** iRoot BP tube showing relatively thick fibrous capsule FC, **c1** the inset showing remodeling of fibrous capsule and collagen fibers (crossed arrow); **d** MTA HP tube showing thin fibrous capsule, **d1** the inset revealing mild pericapsular fibrosis and calcification (arrow). The 3rd panel α-SMA immunostaining within the fibrous capsule, **e** Control tube, **f** ACTIVA tube, **g** iRoot BP tube which showed marked immunostaing and **h** MTA HP tube. Tailed-arrow indicates the expression α-SMA within myofibroblasts. α-SMA IHC. The 4th panel caspase 3 immunostaining within the fibrous capsule, **i** Control tube, **j** ACTIVA tube, **k** iRoot BP tube and **l** MTA HP tube. Wavy-arrow indicates caspase 3 immunostaining within the fibrous capsule. Caspase 3 IHC. TO: Tube Opening, IR: Inflammatory Reaction, FC: Fibrous Capsule

After 4 weeks, control group demonstrated thin mature fibrous capsule with sparse inflammatory reaction and with normal continuity to neighboring fibrous and adipose connective tissues (Fig. 3a and a1). ACTIVA group showed progressive healing with abrogation of coagulative necrosis and inflammatory cells aggregation. A well-formed fibrous capsule was consisted mainly of multiple parallel collagen fibers. In addition, marked decrease of the inflammatory reaction was noticed by regression of lymphocytes and macrophages infiltration (Fig. 3b and b1).

iRoot BP group demonstrated moderate inflammation, slight angiogenesis and mineralization alongside the tube orifice. In addition, marked thickening of the fibrous capsule was still noticeable (Fig. 3c and c1). MTA- HP group showed restoration of the normal tissue. However, limited organization of the fibrous capsule and small tiny particulates of mineral deposition were noticed (Fig. 3d and d1).

### Immunohistochemistry analyses

#### α-SMA antibody

**Fig. 4:**
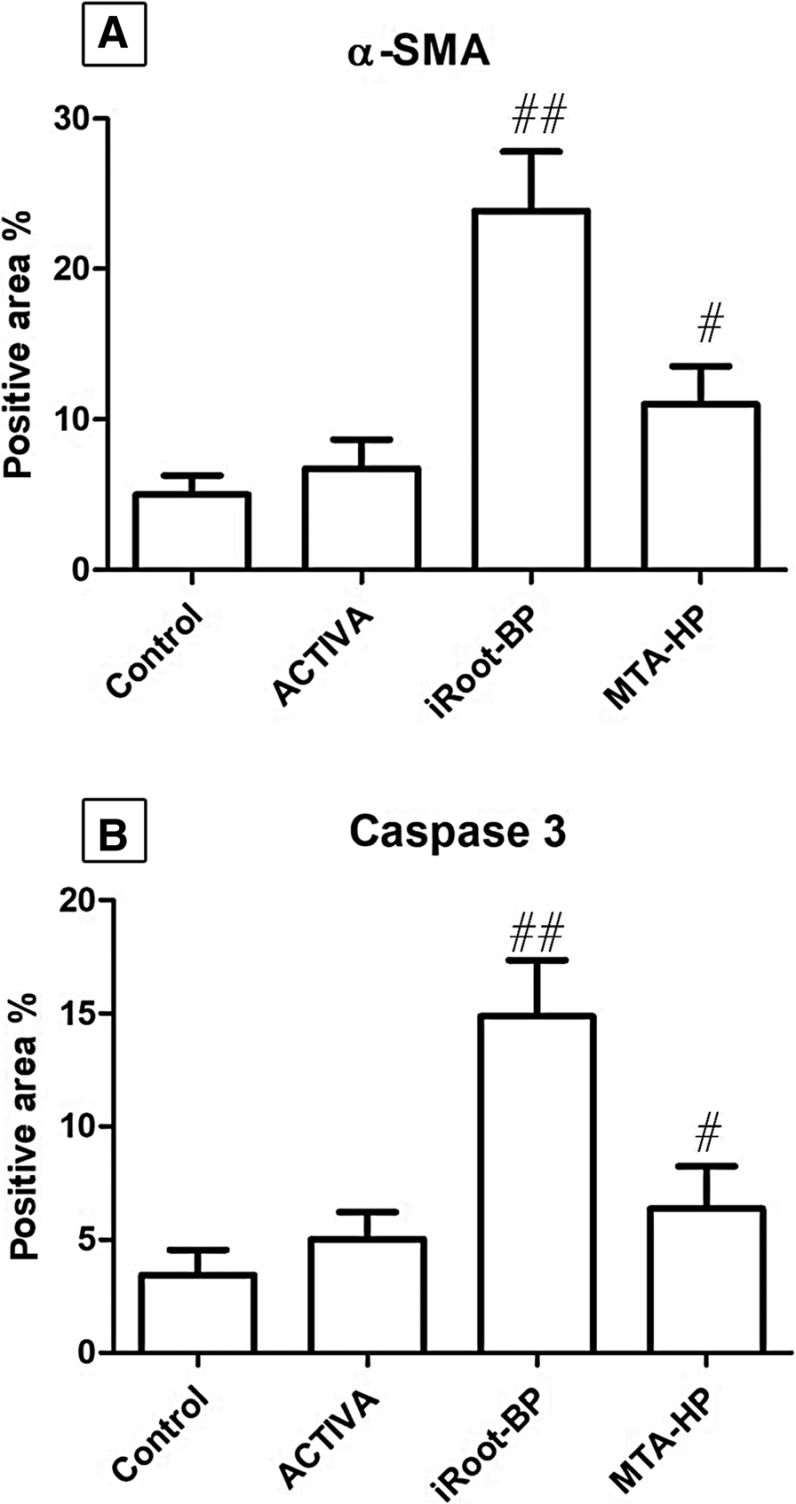
Representative chart of α-SMA (**a**) and caspase 3 (**b**) immunoreaction, data were expressed as percentage of positive area (mean ± SD), # and ## indicate significance at *p* < 0.01 and 0.001 respectively

The α-SMA antibody was expressed mostly within the myofibroblast. Mild expression was seen within the fibrous capsule of the control tube (Fig. 3e). The fibrous capsule around ACTIVA group tube orifice showed similar expression pattern to the control tube (Fig. 3f). Marked expression of α-SMA was noticed within the fibrous capsule around iRoot BP group tube orifice in comparison with the control tube (*p <* 0.05) (Fig. 3g). MTA- HP group tube revealed an increase of positive immunostaining in comparison to the control tube (*p* < 0.05) (Fig. 3h) - (Fig. 4a).

#### Caspase 3 antibody

A positive expression of caspase 3 antibody was noticed mainly within the macrophages and instantly within the fibroblasts. Scanty expression of caspase 3 was noticed within the fibrous capsule around the control tube (Fig. 3i). ACTIVA group tube orifice also showed mild positive reaction (Fig. 3j). Marked increase of caspase 3 positive reaction only appeared within the capsule of iRoot BP group tube in comparison to the control group (*p <* 0.05) (Fig. 3k). The immunoexpression of caspase 3 within MTA- HP group tube was nearly comparable to iRoot BP group (Fig. 3l) - (Fig. 4b).

## Discussion

Minimum toxicity without provoking significant inflammatory response is a fundamental prerequisite of biocompatible materials [22]. Many trials have been undertaken to develop materials with comparable bioactive properties to that of calcium silicate-based materials. These efforts resulted in emergence of both ACTIVA and TheraCal (Bisco Inc., Schamburg, IL, USA). TheraCal is a light-cured calcium silicate-based liner developed for pulp capping procedures with proven successful biological outcomes [23–26]. On the other hand, ACTIVA is considered as RMGI, according to the FDA document [27] (Accessed at 13th of June 2019) and has similar bioactivity and biocompatibility as TheraCal, and can also be used as base material (dentine substitute). Most of the endodontic materials are liable to be used in close contact with human tissues in case of perforations, apical barriers and retrograde fillings. Therefore, these materials must be evaluated carefully in the terms of cytotoxicity and biocompatibility to ensure success of the treatment [28].

Two methods are available to assess the biocompatibility, including in vitro studies such as cytotoxicity using cell culture, genotoxicity and in vivo studies including bone implant and subcutaneous tissue surgical placement. Subcutaneous surgical placement of polyethylene tube containing testing material, has been advocated for testing their biocompatibility and the tissue reaction [29]. The similarity between root canal condition and extruded material at the orifice of polyethene tube creates subcutaneous tissue-tube interface which triggers an inflammatory response with adsorption of tissue fluids and blood into the tube site [30].

Subcutaneous surgical placement of ACTIVA resulted in fast healing with moderate pleomorphic nuclear leukocytes (PMNs), lymphocytes infiltration, early granulation tissue formation and a well formed medium fibrous capsule after a 4 week post-implantation similar to the control group. Biocompatibility of resin containing materials is highly affected by the volume and nature of its leachable elements [31]. Hence the good results of ACTIVA might be due to absence of bisphenol A, Bis-GMA and BPA derivatives which are well known as a serious hazard and aetiologies to irreversible effects on living cells [32]. These results were consistent with the findings [24, 25] with TheraCal showing superior biocompatibility and stimulating more osteoblast-like cells growth and differentiation compared to MTA.

Meanwhile, iRoot BP tubes revealed an initial intense inflammation with an appearance of multi nuclear giant cells (MNGCs) and extensive fibrosis with evidence of bio mineralization and mineral deposition, which was extended untill the 4th week and a thick capsular formation was observed. Furthermore, the MTA-HP implant demonstrated similar chronic nature of tissue response with bio mineralization activity also, but with a lesser degree and a smaller capsular formation than that observed with iRoot BP. These findings were supported by previous studies [33, 34] who reported that both MTA Angelus and iRoot BP exhibited higher fibroblast induction and production of bioactive molecules in comparison to ProRoot MTA .

White MTA-HP had better biocompatibility and wound healing in comparison to iRoot BP and did not induce significant necrosis or apoptosis [35]. Removal of bismuth oxide from the constituents of MTA HP significantly reduced the cytotoxicity affecting osteoblasts and dental pulp cells, particularly when combined with dicalcium silicate [36].

Also, iRoot BP was reported to reduce the cell viability of osteoblast cells in comparison to MTA [12, 37]. And similarly, another study reported that higher concentrations of MTA suppressed cell viability and sialoprotein production of cementoblasts [38]. In addition, high alkalinity of MTA initially induced severe inflammation associated with coagulative necrosis and dystrophic calcifications within superficial cell layers of adjacent connective tissues [6, 39].

Different phases of Calcium silicate hydrate are formed by hydration during a mixing process [40]. Moisture severely affects the bioactivity of the cement starting from the nucleation process of calcium ions until the formation of portlandite occurs. The continuous outflux of calcium hydroxide from CSH helps portlandite crystals to nucleate inside the hydrating wet paste. Calcium phosphate and apatite deposit on cement surfaces as a result of exposure to tissue fluids after being placed subcutaneously [4, 41].

The hydrophobic nature of resin components incorporated in light-cured calcium silicate-based materials decreases its solubility compared to MTA. The resin matrix contains pores which allow water penetration in both in and out directions, thus justifying their ability of calcium ions solubilization and formation of calcium silicate hydrate and portlandite [25, 42, 43].

In the study by Zamparini et al. [44] ready-made paste bioceramic materials containing tantalum pentoxide as radiopacifier showed higher calcium and silicate ions release. In addition there is a direct correlation between calcium ions released from such materials and the proliferation and differentiation of pluripotent-mesenchymal cells into osteoblast like cells regardless the pH level [45].

The contact between the retrograde filling materials and subcutaneous tissue cells induce injury. The healing process within the fibrous capsule depends mainly on the myofibroblast-positive α-SMA. Alpha-SMA is a mechanosensitive protein, that has a direct role in β- cytoplasmic actin stress fibers deposition and improving fibroblast contraction after granulation tissue formation [46]. The decreased α-SMA expression level observed in control and ACTIVA groups was related to less fibrosis formation in comparison with iRoot BP and MTA HP groups that showed intense fibrous reaction.

Apoptosis has a principal role in the repair process mainly in removal of granulation tissue and inflammatory cells which mostly mediate through caspase enzymes. Caspases are a cysteine-requiring aspartate protease that are critically involved in this process. Interestingly, the ACTIVA revealed a marked decrease caspase 3 expression. However, a significant increase in caspase 3 expression in iRoot BP and MTA-HP groups could indicate the ongoing tissue damage. It is noteworthy that many myofibroblast showed apoptosis-like cell death mechanisms in the late stage of wound healing, which might be responsible for disappearance of myofibroblast [47]. Thus, the higher expression index of caspase 3 in the iRoot BP and MTA-HP may indicate a delayed maturation phase of capsule healing consistent with still present myofibroblasts within the fibrous capsule [48].

Periapical area after root end surgery exhibit a challenging environment for retrograde filling materials due to the presence of moisture, blood contamination, bone defects and difficult accessibility. Excessive moisture or blood contamination will have an adverse effect on the mechanical properties and sealing abilities of RMGI and calcium silicate- based materials although; the later use moisture/water for their setting [49, 50]. Occasional challenges faced with MTA represented by the difficulties in manipulation and long setting time increase the possibility of dislocation by irrigating solutions or early occlusal loading [51]. Nevertheless the bioactive RMGI showed promising outcome in previous studies this may be attributed to the bioactivity of this material and high reactivity towards water which plays a great role in deposition of crystals in the exposure sites [52].

## Conclusion

In light of the results of this study, it is concluded that a new bioactive RMGI (ACTIVA) exhibited excellent biocompatibility and healing ability for rat subcutaneous tissues in comparison with calcium silicate- based cements. Therefore, further studies for the biological and mechanical properties of this new bioactive RMGI are needed.

## Data Availability

All data generated or analyzed during this study are included in this published article.

## Abbreviations

IR: Inflammatory reaction
MNGCs: Multi nuclear Giant cells
PMNs: Pleomorphic nuclear leukocytes
RMGI: Resin modified glass ionomer
